# Supplementary material for: Antitumor and Antibacterial Activity of Ni(II), Cu(II), Ag(I), and Hg(II) Complexes with Ligand Derived from Thiosemicarbazones: Characterization and Theoretical Studies
Source: Molecules. 2023 Mar 13;28(6):2590. doi: 10.3390/molecules28062590 (PMC10058203; doi:10.3390/molecules28062590)
Supplement: Supplementary file 1 [file molecules-28-02590-s001.zip › molecules-2254899-supplementary.pdf]

Section S1: Material and physical measurements

All organic compounds and solvents were purchased from Fluka or Merck, Naser City, Egypt. The metal salts $NiCO_3$, $Cu(ClO_4)_2$, Ag_2S, and HgI_2, were obtained from Fluka and utilized for complex synthesis without further purification. Elemental analyses (C, H and N) were carried out at the Microanalytical Unit, Cairo University. Metal content was estimated complexometric using EDTA following standard literature methods. The Fourier Transform Infrared (FT-IR) measurements were performed (4000–400 cm^{-1}) in the KBr discussion. Nenexeus-Nicolidite-640-MSAFT-IR. Mass spectra were acquired using the electron impact (EI) ionization technique at 70 eV on a Hewlett–Packard MS-5988 GC–MS instrument at the Microanalytical Center, National Center for Research, Egypt. The UV–visible absorption spectra were measured in DMF solution (10^{-3} M) using a 4802 UV/vis double beam spectrophotometer. In DMF solution (10^{-3} M) using a Tacussel conductometer type CD6N the molar conductivity measurements were made. The magnetic properties of the complexes were measured at room temperature by the modified Gouy method using Magnetic Susceptibility Johnson Matthey Balance. The effective magnetic moments were calculated using the relation $\mu eff = 2.828(X_mT)^{1/2}$ B.M., where X_m is the molar magnetic susceptibility corrected for diamagnetism of all atoms in the compounds using Selwood and Pascal's constants. Thermal analysis (TGA/DTG) was obtained out by using a Shimadzu DTG-50 Thermal analyzer with a heating rate of 10 Cº/min in a nitrogen atmosphere with the following rate 20 ml/min in the temperature range 25–800 Cº using platinum crucibles at Central Lab, Faculty of Science, Menoufia University, Egypt. By using Rigku Model ROTAFLEX Ru-200 X-ray diffractograms of solid samples were measured at The National Research Center, Cairo, Egypt, by PHILIPS_MPDX′PERT X-ray diffractometer ready with Cu radiation Cu Ka (k = 1.540 56 A °) and structural analysis by x-ray diffractograms give computer control formally was finished. Most powder diffractometers use Bragg-Brentano parafocusing geometry. The X-ray tube was utilized for a copper tube operating at 40 kV and 30 mA. The scanning range (2 ϴ) was 20–80º with a step size of 0.02º and a counting time of 3 s/step. Quartz was utilized as the standard material to precise for the instrumental extension This identification of the complexes was done by a known method from the fit identified Scherrer formula, the average crystallite size, L, is L = ƛK=ßcosϴ. Where ƛ is the X-ray wavelength in the manometer, K (constant equal to 0.9) is related to crystallite shape, and ß is the peak width at half maximum height. The value of ß in the 2ϴ axis of diffraction shape must be in radians. The ϴ is the Bragg angle and is able to in radians since the Cos ϴ compatible with the same number.

Section S2. Computational study

All calculations were performed using Gaussian 09 rev. A.02 (Wallingford, CT,2009) by the DFT/B3LYP method. 6/311G and LANL2DZ are the standard basis sets for the synthesized ligand and its metal complexes, respectively. MOE 2009 (Molecular Operating Environment) software simulates the topoisomerase IIa. The protein crystal structure of the protein with topoisomerase II DNA gyrase enzymes (antimicrobial activity) (PDB ID: 2XCT)

Section S3. Antibacterial screening studies

A bacterial suspension was prepared for each strain according to 0.5 McFarland turbidity standards. All strains were streaked on *Mueller–Hinton agar* medium to test the antimicrobial activity of synthesized compounds. Dimethylsulfoxide (DMSO) was used as the solvent for synthesized compounds. Wells with a 8 mm diameter were filled with 100 μl of each of the different synthesized compounds and incubated aerobically at 37°C for 24 hours. A prepared solution of ampicillin and gentamicin with concentration of 1 mg/ml (Oxoid, UK) were utilized as positive antimicrobial standards for gram-positive and gram-negative bacteria respectively. DMSO was used as negative control. All test samples and standards were performed in triplicate. The clear zone, which represents the organisms' inhibition, was measured and then used to determine the mean of the zones of inhibition.

Section S4. Cytotoxicity Assays

The MTT assay protocol was carried out as follows: The 96 well tissue culture plate was implanted with 1 X 105 cells / ml (100 ul / well) and cultured at 37 degrees Celsius for 24 hours to form a full monolayer sheet. After forming a confluent sheet of cells, growth medium was drained from 96 well micro titer plates, and the cell monolayer was washed twice with wash media. Two-fold dilutions of the tested samples were prepared in RPMI medium containing 2% serum (maintenance medium). In each well, 0.1 ml of each dilution was assayed, with three wells serving as controls and receiving only maintenance media. The plate was incubated at 37 degrees Celsius and Cells were examined for any physical evidence of toxicity, such as partial or total monolayer loss, rounding, shrinkage, or cell granulation. MTT solution (5mg/ml in PBS) was prepared (BIO BASIC CANADA INC). Each well received a 20ul MTT solution. Shake the MTT into the medium for 5 minutes at 150rpm on a shaking table. Incubate for 1-5 hours (37°C, 5% CO_2) to allow the MTT to be metabolized. Discard the media.(If required, dry plate on paper towels to remove residue. In 200ul DMSO, resuspend formazan (MTT metabolic product).Shake at 150rpm for 5 minutes to properly mix the formazan into the solvent. At 560nm, measure optical density and extract background at 620nm.The optical density should be proportional to the number of cells.

Table S1. The optimized bond lengths, Å, and bond angles, degrees for the synthesized ligand and complexes based on B3LYP/6-311G and B3LYP/LANL2DZ, respectively.

Bond length (Ao)	H_2L	1S	2S	3S	4S
R(Ni-S14)	---	2.43517	---	---	---
R(M-N12)	---	2.04567	2.13926	2.27455	2.15081
R(Ni-O24)	---	1.85452	---	---	---
R(Ni-O26)	---	1.84706	---	---	---
R(M-O10)	---	2.81738	1.91503	---	2.05794
R(Ag23-S14)	---	---	---	2.91386	---
R(Ag25-S14)	---	---	---	2.76721	---
R(Ag23-S24)	---	---	---	2.43967	---
R(Ag25-S24)	---	---	---	2.58991	---
R(Ag23-O10)	---	---	---	2.43537	---
R(Ag25-O26)	---	---	---	2.39659	---
R(Hg-I)	---	---	---	---	1.90173
R(N15-C16)	1.42522	1.44487	1.45113	1.42006	1.41825
R(C13-N15)	1.37324	1.35019	1.35817	1.37963	1.44553
R(N12-C13)	1.35973	1.6043	1.44088	1.45488	1.48604
R(N11-N12)	1.39358	1.42010	1.49543	1.44784	1.41808
R(C9-N11)	1.35644	1.39309	1.32355	1.40508	1.34556
R(O10-C9)	1.25807	1.24676	1.33177	1.28210	1.26576
R(C8-C9)	1.51805	1.53217	1.51589	1.52930	1.51228
R(C8-N7)	1.44044	1.46272	1.45124	1.44507	1.45123
R(C13-S14)	1.73102	1.72206	1.71600	1.78215	1.83130
A(C16-N15-C13)	128.843	125.824	129.048	130.973	122.788
A(N15-C13-Nl2)	112.689	120.059	117.302	118.301	108.876

A(C13-N12-Nl1)	121.716	119.256	114.538	124.561	107.191
A(N12-Nl1-C9)	117.735	118.081	110.556	116.849	120.277
A(O10-C9-N11)	120.412	122.004	125.654	122.169	117.933
A(O10-C9-C8)	123.293	123.636	116.308	123.314	122.367
A(Nl1-C9-C8)	116.294	114.290	118.038	114.510	119.699
A(C9-C8-N7)	108.574	114.014	111.935	110.274	112.277
A(O24-C25-O26)	---	104.286	---	---	---
A(O24-S14-C13)	---	104.154	---	---	---
A(O24-O10-S14)	---	55.550	---	---	---
A(O26-Ni-S14)	---	97.622	---	---	---
A(O10-Cu-O33)	---	---	160.487	---	---
A(N12-M-N35)	---	---	141.003	---	122.604
A(O33-N11-C9)	---	---	84.177	---	---
A(Ag23-S14-Ag25)	---	---	---	74.087	---
A(S14-Ag25-O26)	---	---	---	92.803	---
A(O10-Ag23-S24)	---	---	---	127.703	---
A(I-Hg-O35)	---	---	---	---	75.281

Where:.
1S=Ni (II) complex.
2S= Cu (II) complex.
3S=Ag (I) complex.
4S= Hg (II) complex.

Table S2. Results of antibacterial activity of ligand (H_2L) and Ni (II), Cu(II), Ag(I), and Hg(II) complexes against different gram-negative and gram-positive bacteria.

Sample<br><br>Microorganism	H_2L	1S<br>Ni(II)comple<br>x	2S<br>Cu(II)comple<br>x	3S<br>Ag(II)comple<br>x	4S<br>Hg(II)comple<br>x	Standard antibiotic
Gram-negative bacteria						Gentamicin
Escherichia coli (ATCC:10536)	15.3±0.6	65.95±0.5	17.6±0.5	19.02±0.6	23.6±0.6	27±0.5
Klebsiella pneumonia (ATCC:10031)	15.3±0.5	57.36±0.6	16.4 ±0.6	18.6±0.6	22.3±0.6	25±0.5
Gram-positive bacteria						Ampicillin
Staphylococcus aureus (ATCC:13565)	13.6±0.5	69.21±0.6	16.6±0.6	15.6±0.5	23.3±0.6	22±0.1
Streptococcus mutans (ATCC:25175)	NA	72.34±0.5	10.6±0.5	20.3±0.6	44.3±0.6	30±0.5

Table S3. Results of cytotoxic activity (IC_{50}) of the ligand complexes with Ni(II), Cu(II), Ag(I), and Hg(II) against human HepG2 cell line at different concentration levels.

No	Compounds	IC_{50} (μM)
H_2L	$C_{15}H_{15}ClN_4OS$	20.45
1S	$C_{16}H_{17}ClN_4NiO_5S$	41.2
2S	$C_{30}H_{29}Cl_2CuN_8O_2S2$	182.61
3S	$C_{15}H_{19}Ag_2ClN_4O_3S2$	293.95
4S	$C_{15}H_{20}ClHgIN_4O_4S$	48.5
Vinblastine		4.58

Table S4. The binding affinity of compounds against a topoisomerase IIa enzyme (code: 2xct).

docking 2xct			
Compound	The energy scoring (RMSD)	Amino acids interacted	Interaction type
H_2L	-4.05(1.81)	Leu-A624 and Lys-A518	Backbone donor and arene cation
1S	-4.46(1.99)	Asn-A553, Gln-A521, and Phe-A642	Backbone donor, Sidechain acceptor, and arene-arene
2S	-8.03(1.44)	Lys-A525, (Asp-A623& Asn-B508) and Gln-B549	Arene-cation, Sidechain acceptor, and Sidechain donor
3S	-3.97(2.69)	Lys-A518 and Asn-B508	Arene-cation and Sidechain acceptor
4S	-3.57(1.76)	Asn-B508	Sidechain acceptor

Table S5. The binding affinity of compounds against EGFR tyrosine kinase receptor (code: 1m17)

docking 1m17			
Compound	The energy scoring (RMSD)	Amino acids interacted	Interaction type
ligand	-5.97(1.51)	---	---
1S	-5.43(2.72)	Lys-721	Side chain donor
2S	-6.08(4.40)	Lys-721 and Asp-831	Arene-cation and Sidechain acceptor
3S	-5.84(2.98)	---	---
4S	-6.88(1.17)	Asp-833, Glu-718, and Lys-721	metal contact interactions

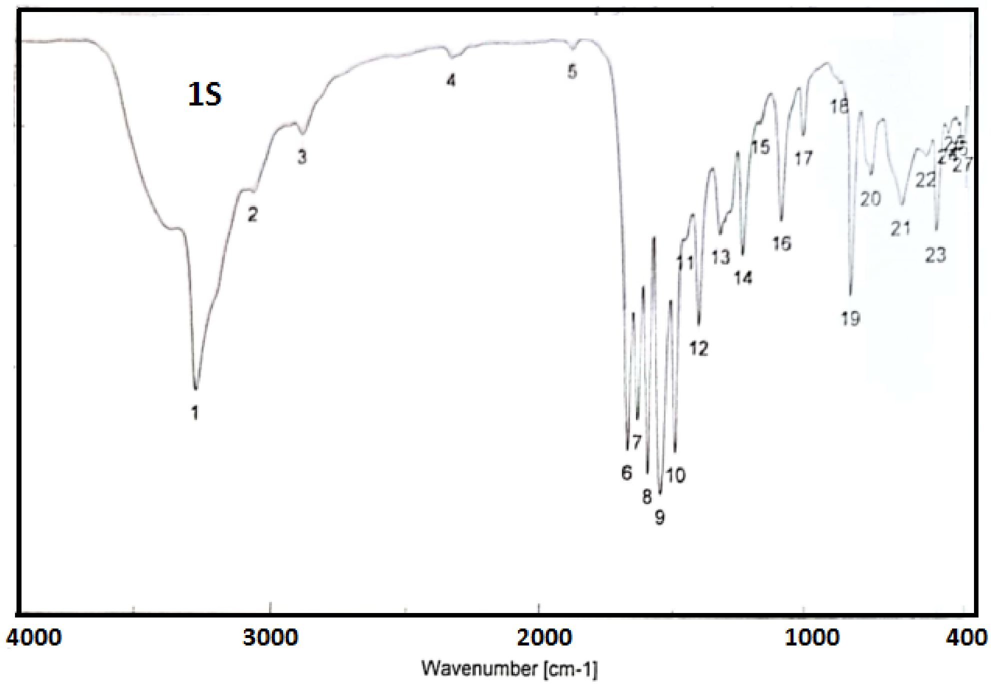

Figure S1 FT-IR spectra of the Ni(II) complexes at 4000 – 400 cm^{-1}

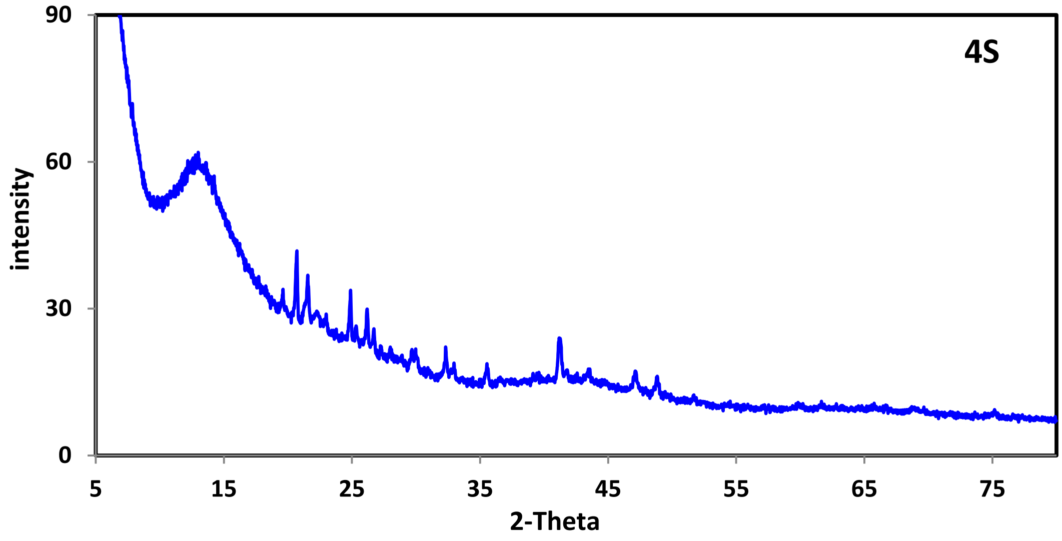

Figure S2 Powder x-ray diffraction graphs for Hg(II) complex (4S)

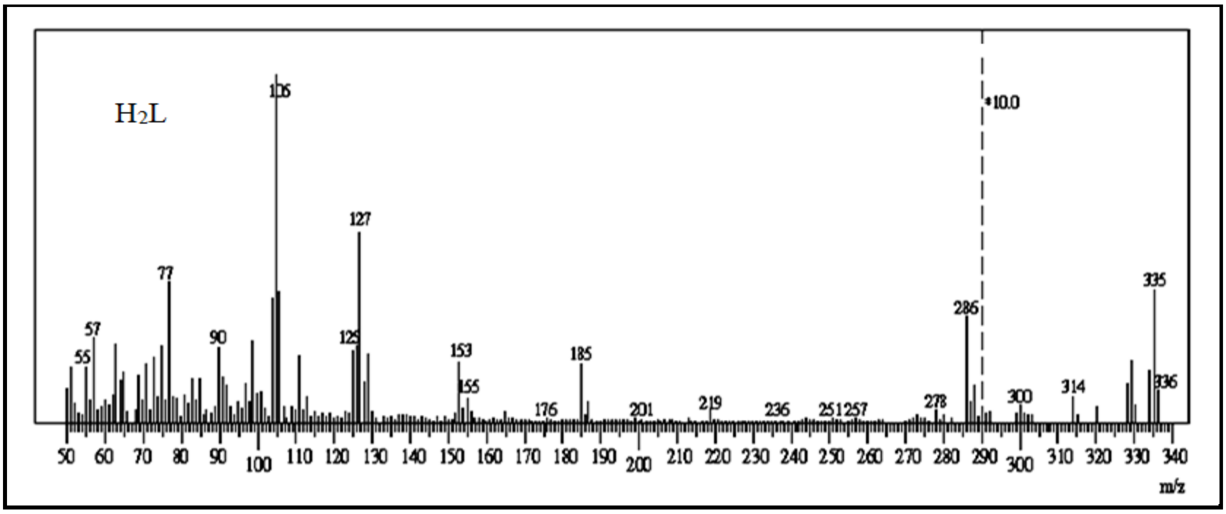

Figure S3. Mass spectrum of the ligand(H_2L)

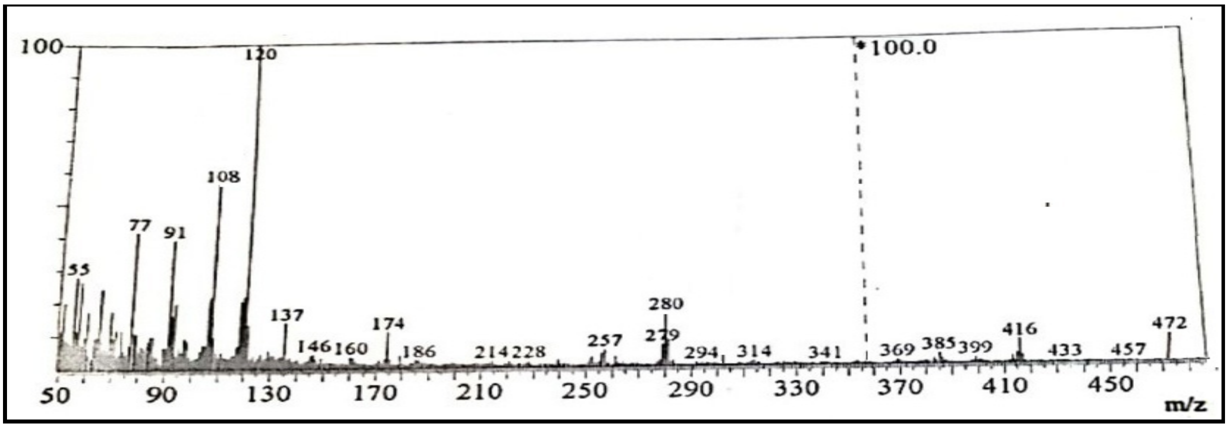

Figure S4 Mass spectra of the Ni(II) complex (1S)

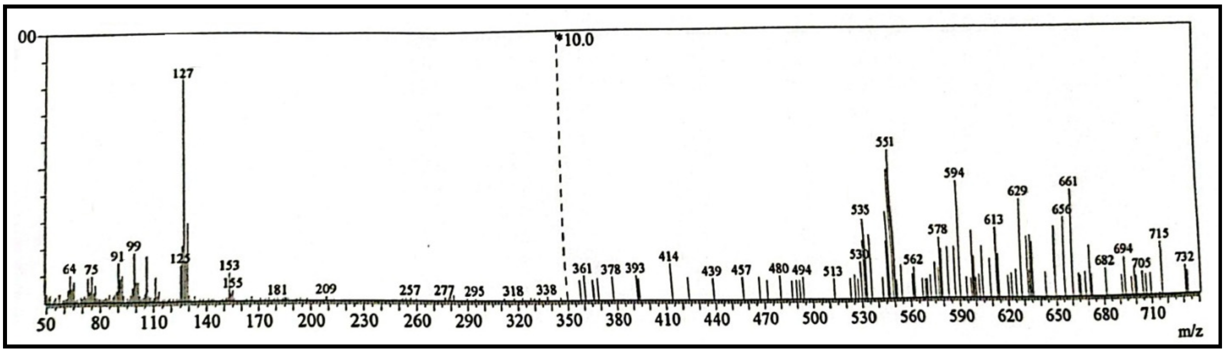

Figure S5 Mass spectra of the Cu(II) complex (2S)

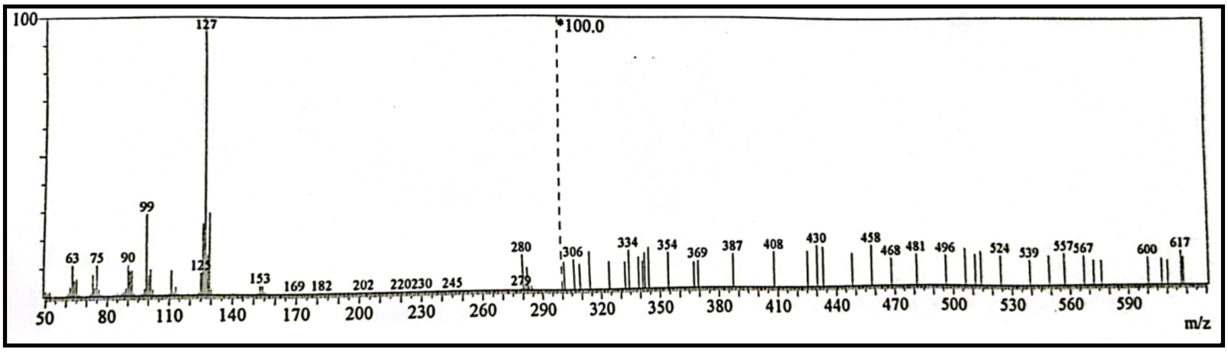

Figure S6 Mass spectra of the Ag(I) complex (3S)

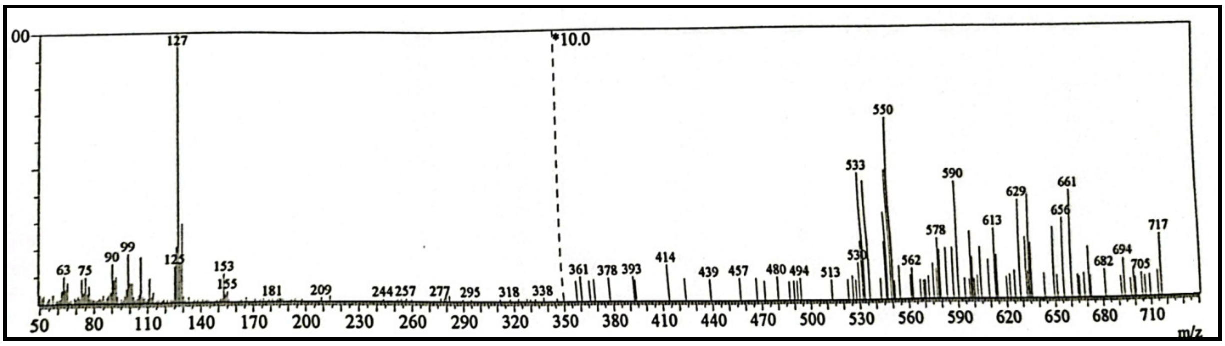

Figure S7 Mass spectra of the Hg(II) complex (4S)
